# Occult Necrotizing Fasciitis Presenting as a Severe Hyperosmolar Hyperglycemic State in a Patient With Newly Diagnosed Type 2 Diabetes Mellitus

**DOI:** 10.7759/cureus.110616

**Published:** 2026-06-10

**Authors:** Daniel Balderrama

**Affiliations:** 1 Internal Medicine, Hospital Metropolitano, Monterrey, MEX

**Keywords:** hyperosmolar hyperglycemic syndrome, necrotizing fasciitis, severe sepsis, soft tissue infections, type 2 diabetes mellitus

## Abstract

Necrotizing fasciitis is a rapidly progressive and life-threatening soft tissue infection in which early diagnosis remains challenging when initial clinical findings are subtle or nonspecific. Hyperglycemic crises are frequently triggered by infection and may obscure the underlying etiology when metabolic abnormalities dominate the initial presentation. We report a case of a 52-year-old female with rheumatoid arthritis receiving chronic corticosteroid therapy, an important risk factor for atypical infectious presentations, who presented with altered mental status, dehydration, and severe hyperglycemia consistent with hyperosmolar hyperglycemic state in the setting of newly diagnosed type 2 diabetes mellitus. Initial evaluation revealed no clear infectious source aside from mild localized edema, and the patient was treated with intravenous fluids, insulin infusion, and broad-spectrum antibiotics. Despite early biochemical improvement, she developed persistent hyperlactatemia, refractory hypotension, and progressive lower extremity swelling with subsequent crepitus. Imaging revealed extensive gas within the deep fascial planes consistent with necrotizing fasciitis, requiring emergent surgical intervention. The patient improved following definitive management and was ultimately discharged. This case highlights the importance of continuous clinical reassessment and maintaining a high index of suspicion for occult infections in patients with hyperglycemic crises who fail to improve as expected.

## Introduction

Hyperglycemic crises remain among the most severe acute complications of diabetes and continue to be associated with significant morbidity and mortality despite advances in recognition and management. Traditionally classified as diabetic ketoacidosis (DKA) and hyperosmolar hyperglycemic state (HHS), these conditions are now better understood as part of a continuum characterized by varying degrees of insulin deficiency, hyperglycemia, and metabolic derangement. In clinical practice, many patients present with overlapping biochemical and clinical features, limiting the utility of rigid diagnostic categories and reinforcing the need for an integrated approach to management [1,2].

Infection is the most common precipitating factor for hyperglycemic crises and has been reported as the major precipitating factor in 30%-60% of patients with HHS [1]. While common infections such as pneumonia and urinary tract infections are often readily identified, deeper or occult sources may initially go unnoticed, particularly in patients with impaired immune responses. Chronic corticosteroid use can attenuate inflammatory signs and delay recognition of infection, allowing disease progression before diagnosis [1].

Necrotizing fasciitis is a rapidly progressive and life-threatening soft tissue infection characterized by extensive fascial necrosis, systemic toxicity, and a high risk of septic shock and death if not promptly treated [3,4]. Early diagnosis remains challenging because initial clinical findings may be subtle, nonspecific, or even misleading, especially in patients with diabetes or immunosuppression [5,6]. Pain out of proportion to physical examination findings is often considered an important early clinical clue and may precede the appearance of more characteristic manifestations [5,6]. Classic features such as crepitus, skin necrosis, and bullae often appear late in the disease course, which may lead to underrecognition during early stages.

An important clinical scenario arises when severe infections present primarily through metabolic decompensation rather than localized symptoms. In such cases, clinicians appropriately prioritize correction of hyperglycemia and fluid deficits; however, failure to achieve expected clinical improvement should prompt reconsideration of the diagnosis and a renewed search for an underlying source of infection [1].

In this context, we report a case of occult necrotizing fasciitis presenting initially as HHS in a patient with newly diagnosed type 2 diabetes mellitus and chronic corticosteroid use, highlighting the importance of continuous clinical reassessment when the clinical course deviates from the expected trajectory after initial metabolic stabilization.

## Case presentation

A 52-year-old female with a history of rheumatoid arthritis treated with chronic prednisone therapy and remote hip prosthesis placement presented with three days of progressive asthenia, adynamia, and altered mental status characterized by visual hallucinations. One day prior to admission, she developed chills and palpitations. She denied fever, gastrointestinal symptoms, urinary complaints, or respiratory symptoms.

On arrival, capillary blood glucose was greater than 500 mg/dL. Physical examination revealed a somnolent patient oriented only to person, disoriented to time and place, with blood pressure of 90/60 mmHg, heart rate of 115 beats per minute, respiratory rate of 37 to 42 breaths per minute, oxygen saturation of 96% on room air, and signs of mild dehydration. She was afebrile. Cardiopulmonary and abdominal examinations were unremarkable. Initial musculoskeletal examination revealed mild edema of the right knee without erythema, warmth, tenderness, or crepitus. The patient reported minimal pain, with preserved range of motion and no overlying skin discoloration or other findings suggestive of a severe soft tissue infection.

Laboratory evaluation demonstrated severe hyperglycemia (630 mg/dL), glycated hemoglobin of 12.9%, venous pH of 7.36, bicarbonate of 19.8 mmol/L, sodium of 130 mmol/L (corrected sodium = 138.5 mmol/L), potassium of 3.0 mmol/L, lactate of 6.3 mmol/L, creatinine of 1.4 mg/dL, and C-reactive protein of 322.7 mg/L. The calculated anion gap was 25.2 mEq/L. Urinary ketones were negative, and serum ketone levels were not available. Effective serum osmolality was approximately 312 mOsm/kg. Although the anion gap was elevated, the absence of significant acidemia and negative ketones made diabetic ketoacidosis unlikely, and the overall findings were more consistent with hyperosmolar hyperglycemic state, with the elevated anion gap likely attributable to severe hyperlactatemia. Initial laboratory findings are summarized in Table 1.

**Table 1:** Laboratory findings during the initial evaluation. * Effective osmolality was calculated using corrected sodium (Na): 2 × Na (corrected) + glucose/18. HbA1c: glycated hemoglobin; pCO_2_: partial pressure of carbon dioxide.

Parameter	Value	Reference range	Interpretation
Glucose	630 mg/dL	70-100 mg/dL	Severe hyperglycemia
HbA1c	12.9%	<5.7%	Chronic uncontrolled diabetes
pH (venous)	7.36	7.35-7.45	No significant acidosis
pCO₂	35 mmHg	35-45 mmHg	Near normal
Bicarbonate	19.8 mmol/L	22-28 mmol/L	Mild metabolic disturbance
Base excess	-5.6	-2 to +2	Mild metabolic acidosis
Anion gap	25.2 mEq/L	8-12 mEq/L	Elevated, likely related to hyperlactatemia
Sodium	130 mmol/L	135-145 mmol/L	Pseudohyponatremia
Corrected sodium	138.5 mmol/L	135-145 mmol/L	Normal after correction
Potassium	3.0 mmol/L	3.5-5.0 mmol/L	Hypokalemia
Chloride	85 mmol/L	98-106 mmol/L	Hypochloremia
Lactate	6.3 mmol/L	<2 mmol/L	Significant hyperlactatemia
Creatinine	1.4 mg/dL	0.6-1.2 mg/dL	Mild acute kidney injury
Blood urea nitrogen (BUN)	24 mg/dL	7-20 mg/dL	Mild elevation
Urea	51.3 mg/dL	15-40 mg/dL	Elevated
C-reactive protein	322.7 mg/L	<5 mg/L	Severe inflammatory response
Hemoglobin	11.27 g/dL	12-16 g/dL	Mild anemia
Leukocytes	6.55 ×10³/µL	4-10 ×10³/µL	Within normal range
Neutrophils	5.44 ×10³/µL	2-7 ×10³/µL	Normal-high
Platelets	191 ×10³/µL	150-400 ×10³/µL	Normal
Urinary ketones	Negative	Negative	No significant ketosis
Effective osmolality*	~312 mOsm/kg	<295 mOsm/kg	Hyperosmolar state

Blood and urine cultures were obtained prior to initiation of antibiotic therapy and subsequently resulted negative. A chest radiograph was obtained during the initial evaluation; however, the image was not available for review at the time of manuscript preparation and did not suggest a clear pulmonary source of infection.

During the first 12 hours of treatment, hyperglycemia improved; however, the patient remained lethargic with persistent hyperlactatemia and progressive hypotension refractory to fluid resuscitation, ultimately requiring vasopressor support. The persistence of elevated lactate despite initial resuscitation raised concern for ongoing tissue hypoperfusion or an occult infectious process rather than isolated metabolic derangement.

Over the following hours, the patient developed progressive swelling of the right lower extremity extending toward the inguinal region. Repeat physical examination revealed new palpable crepitus along the affected extremity. Concurrently, the patient developed high-grade fever with temperatures up to 40°C.

Contrast-enhanced computed tomography of the pelvis and right lower extremity demonstrated extensive gas within the deep fascial planes (Figure 1), with further extension toward the inguinal region (Figure 2), findings consistent with necrotizing fasciitis.

**Figure 1:**
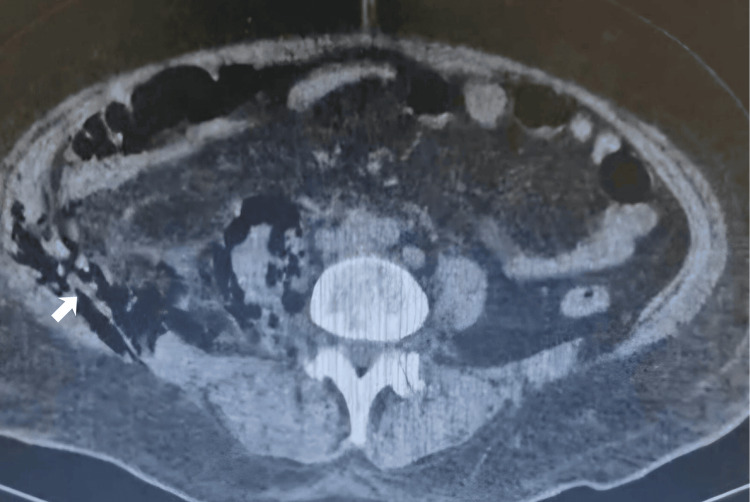
Computed tomography of the pelvis and right lower extremity (axial view). Axial contrast-enhanced computed tomography demonstrating multiple foci of gas within the deep fascial planes and adjacent soft tissues of the right lower extremity (arrow), a characteristic imaging finding of necrotizing fasciitis.

**Figure 2:**
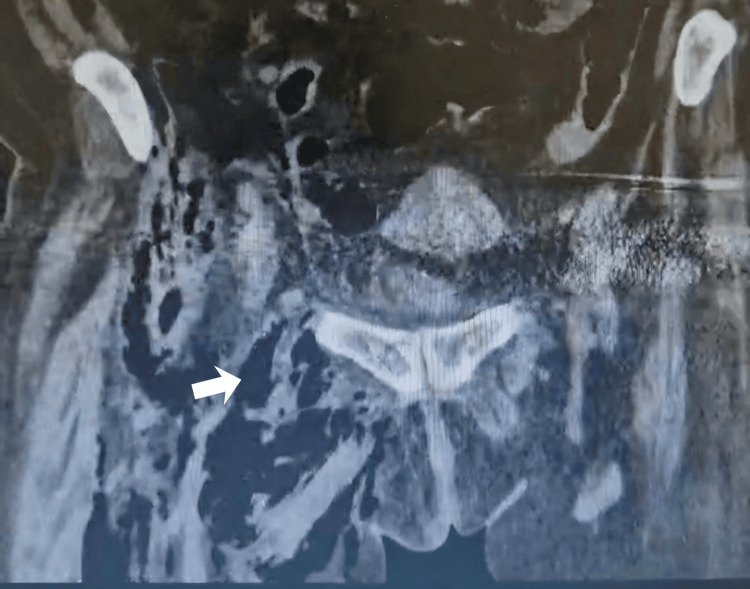
Computed tomography of the pelvis and right lower extremity (coronal view). Coronal contrast-enhanced computed tomography demonstrating extensive gas tracking along the deep fascial planes with extension toward the inguinal region (arrow), indicating widespread soft tissue involvement consistent with necrotizing fasciitis.

Antibiotic therapy was escalated, and emergent surgical consultation was obtained. Due to the extent of infection, the patient underwent urgent right lower extremity disarticulation. Intraoperative tissue specimens were obtained for microbiological analysis and subsequently grew *Streptococcus pyogenes*, confirming the infectious etiology of the necrotizing fasciitis.

Following surgical intervention, the patient demonstrated progressive hemodynamic stabilization, resolution of vasopressor requirements, and improvement in mental status. She was transitioned from intravenous insulin infusion to a subcutaneous insulin regimen and ultimately discharged with outpatient follow-up.

The chronological progression of clinical findings and interventions is summarized in Table 2.

**Table 2:** Clinical timeline and key events during hospitalization.

Timepoint	Clinical findings	Interventions	Clinical significance
Day -3	Asthenia, adynamia, altered mental status	None	Early nonspecific presentation
Admission	Severe hyperglycemia (>500 mg/dL), dehydration, and altered mental status	IV fluids, insulin infusion initiated	Hyperglycemic crisis suspected
Admission (early)	No clear infectious source identified	Empiric broad-spectrum antibiotics initiated (meropenem)	Infection suspected despite unclear source
0-12 hours	Improvement in glucose levels	Continued fluids, insulin, electrolyte replacement	Partial metabolic stabilization
~12 hours	Persistent hyperlactatemia, encephalopathy	Ongoing resuscitation	Suggests an unresolved underlying process
18-24 hours	Hypotension refractory to fluids	Vasopressor support initiated	Hemodynamic deterioration
~24 hours	Progressive right lower extremity swelling	Clinical reassessment	Emerging localizing signs
Shortly after	Palpable crepitus detected	Urgent imaging requested	Suspicion for necrotizing infection
Same day	CT: gas in fascial planes	Antibiotics escalated (meropenem + vancomycin + clindamycin)	Confirms necrotizing fasciitis
Emergency	Extensive soft tissue involvement	Surgical disarticulation	Definitive source control
Postoperative	Hemodynamic stabilization, improved mental status	Transition to subcutaneous insulin	Clinical recovery

## Discussion

Hyperglycemic crises represent complex metabolic emergencies in which biochemical abnormalities frequently dominate the initial clinical presentation, sometimes obscuring the underlying precipitating condition. Current evidence supports the concept that diabetic ketoacidosis and hyperosmolar hyperglycemic state exist along a continuum rather than as strictly distinct entities, with many patients demonstrating overlapping clinical and laboratory features [1,2].

Infection remains the most common precipitating factor for hyperglycemic crises; however, identifying the source is not always straightforward. While common infections are often recognized early, deep-seated or rapidly progressive infections may initially lack clear localizing signs, particularly in immunocompromised patients. Chronic corticosteroid use, as in this case, can attenuate inflammatory responses and delay recognition of infection [1].

A key clinical challenge arises when metabolic parameters improve, but the patient fails to demonstrate parallel clinical recovery. In such situations, persistent abnormalities such as ongoing encephalopathy, refractory hypotension, or sustained hyperlactatemia should prompt reconsideration of the initial diagnosis. Although elevated lactate levels may initially be attributed to hypoperfusion related to dehydration, their persistence after adequate resuscitation should raise concern for ongoing tissue hypoxia or sepsis. In this context, persistent hyperlactatemia after apparent metabolic stabilization should not be attributed solely to hyperglycemia and must prompt evaluation for occult infection.

In the present case, persistent hyperlactatemia despite initial correction of hyperglycemia and aggressive fluid resuscitation served as a key warning sign that the patient's clinical deterioration could not be explained solely by metabolic derangement. This finding prompted repeated physical examinations and further diagnostic evaluation, ultimately leading to recognition of progressive lower extremity swelling, the detection of crepitus, and subsequent cross-sectional imaging that confirmed necrotizing fasciitis.

Necrotizing fasciitis represents one of the most severe and rapidly progressive infectious processes, characterized by extensive fascial necrosis, systemic toxicity, and high mortality, with reported mortality rates ranging from 20% to 40% [3,4]. Early diagnosis remains challenging because initial manifestations may be subtle, nonspecific, or even misleading, particularly in patients with diabetes or immunosuppression [5,6]. Classic findings such as crepitus, skin necrosis, and bullae often appear late in the disease course and may be absent early.

The progression observed in this patient reflects the typical evolution of necrotizing soft tissue infections when diagnosis is delayed. Imaging plays a crucial role once suspicion arises, with the presence of gas within fascial planes serving as a key diagnostic feature.

The relationship between severe infection and hyperglycemic crises is bidirectional. Infection acts as a potent metabolic stressor through increased counterregulatory hormone activity, precipitating hyperglycemia and metabolic decompensation, while hyperglycemia itself impairs immune function and promotes bacterial proliferation, facilitating rapid disease progression [7].

Previous reports have described similar cases in which necrotizing soft tissue infections initially present with metabolic crises, delaying recognition of the underlying pathology [8]. These findings reinforce the need for heightened clinical vigilance when the patient’s clinical course deviates from the expected trajectory.

This case underscores the importance of continuous clinical reassessment in patients with hyperglycemic crises. Repeated physical examination remains one of the most valuable tools in detecting evolving pathology. Failure to improve after appropriate treatment should never be attributed solely to the severity of metabolic derangement, and clinicians must actively search for underlying causes.

This report is limited by its nature as a single case, which may limit the generalizability of the observations. Although the temporal relationship between severe infection and hyperglycemic crisis was evident, causal relationships between the severity of hyperosmolar hyperglycemic state and the progression of necrotizing fasciitis cannot be established. Nevertheless, the diagnosis was supported by characteristic imaging findings, intraoperative assessment, and microbiological confirmation from tissue cultures that grew *Streptococcus pyogenes*. Despite these limitations, this case provides clinically relevant insight into a scenario in which metabolic abnormalities may mask a rapidly progressive and life-threatening infection.

## Conclusions

Hyperglycemic crises should be approached as complex clinical syndromes that frequently reflect an underlying precipitating condition. This case illustrates how severe metabolic abnormalities may obscure a life-threatening infection such as necrotizing fasciitis.

Persistent hyperlactatemia, hemodynamic instability, or lack of expected clinical improvement should prompt immediate reassessment. Early recognition and timely surgical intervention remain critical determinants of survival.
